# The Modulatory Effects of Intermittent Theta Burst Stimulation in Combination With Mirror Hand Motor Training on Functional Connectivity: A Proof-of-Concept Study

**DOI:** 10.3389/fncir.2021.548299

**Published:** 2021-04-29

**Authors:** Jack Jiaqi Zhang, Kenneth N. K. Fong

**Affiliations:** Department of Rehabilitation Sciences, The Hong Kong Polytechnic University, Hung Hom, Hong Kong

**Keywords:** theta burst stimulation, mirror training, mirror visual feedback, coherence, electroencephalogram

## Abstract

Mirror training (MT) is an observation-based motor learning strategy. Intermittent theta burst stimulation (iTBS) is an accelerated form of excitatory repetitive transcranial magnetic stimulation (rTMS) that has been used to enhance the cortical excitability of the motor cortices. This study aims to investigate the combined effects of iTBS with MT on the resting state functional connectivity at alpha frequency band in healthy adults. Eighteen healthy adults were randomized into one of three groups—Group 1: iTBS plus MT, Group 2: iTBS plus sham MT, and Group 3: sham iTBS plus MT. Participants in Groups 1 and 3 observed the mirror illusion of the moving (right) hand in a plain mirror for four consecutive sessions, one session/day, while participants in Group 2 received the same training with a covered mirror. Real or sham iTBS was applied daily over right motor cortex prior to the training. Resting state electroencephalography (EEG) at baseline and post-training was recorded when participants closed their eyes. The mixed-effects model demonstrated a significant interaction effect in the coherence between FC4 and C4 channels, favoring participants in Group 1 over Group 3 (Δβ = −0.84, *p* = 0.048). A similar effect was also found in the coherence between FC3 and FC4 channels favoring Group 1 over Group 3 (Δβ = −0.43, *p* = 0.049). In contrast to sham iTBS combined with MT, iTBS combined with MT may strengthen the functional connectivity between bilateral premotor cortices and ipsilaterally within the motor cortex of the stimulated hemisphere. In contrast to sham MT, real MT, when combined with iTBS, might diminish the connectivity among the contralateral parietal–frontal areas.

## Introduction

Mirror training (MT), in which participants are required to move one side of their hand while simultaneously observing the mirror visual feedback (MVF) from a mirror placed in the mid-sagittal plane, has been investigated with healthy adults to study the process of observation-based motor learning (Zult et al., 2016; Chen et al., 2019) and also applied in stroke rehabilitation to improve the upper extremity motor relearning in patients with hemiplegia (Fong et al., 2019). The neural correlates underlying the MT are still under exploration, and there has been evidence that MVF can activate the parietal–frontal areas across bilateral hemispheres as well as the ipsilateral sensorimotor area (ipsilateral to the training hand and contralateral to the static hand behind the mirror) (Zhang et al., 2018). A possible explanation is that the parietal–frontal area encompasses the so-called human mirror neuron system (MNS), which can be activated during both observation and execution of movements (Rizzolatti and Craighero, 2004). In humans, it is believed that mirror neurons are located in the inferior frontal gyrus and adjacent premotor cortex, and in the rostral part of the inferior parietal lobule (Iacoboni and Dapretto, 2006). The role of the MNS is to facilitate the sensorimotor area in order to prepare the brain to be more receptive and ready to acquire new motor skills, in either healthy individuals (Bahr et al., 2018) or patients with stroke (Ding et al., 2018; Bai et al., 2019b).

Some studies have used non-invasive brain stimulation (NIBS)—most commonly repetitive transcranial magnetic stimulation (rTMS) and transcranial direct current stimulation (tDCS), to modulate the brain response to MT (von Rein et al., 2015; Kim and Yim, 2018; Jin et al., 2019). Facilitating the motor cortex by excitatory NIBS, including high-frequency rTMS or anodal tDCS, prior or concurrently to MT showed a greater effect on enhancing motor performance (measured using a two-ball rotation task) in healthy adults (von Rein et al., 2015) and motor recovery (measured using the box and block test or the action research arm test) in patients with stroke (Kim and Yim, 2018; Jin et al., 2019), indicating a synergistic effect when combining these two treatment modalities. Intermittent theta burst stimulation (iTBS) is an accelerated form of excitatory rTMS as it can yield a similar effect with high-frequency rTMS by using a very short conditioning time (i.e., 600 pulses a session, delivered in 3 min) (Huang and Rothwell, 2004). There is evidence to support that iTBS delivered to the motor cortex could enhance the efficacy of motor training in healthy individuals (Platz et al., 2018) and patients with stroke (Ackerley et al., 2016). However, it is still unclear whether iTBS could enhance observation-based motor learning via MT, and what its underpinning neural correlates are.

Brain activation during MVF training has been investigated in previous studies. Studies have shown the activation of the ipsilateral motor cortex (ipsilateral to the training hand and contralateral to the MVF) caused by MT (Saleh et al., 2017; Bai et al., 2019a). The activation of frontal regions, for example, premotor area, and parietal regions, has been also reported in previous literature (Hamzei et al., 2012; Saleh et al., 2017; Bai et al., 2019a; Ding et al., 2019). However, the intercortical functional connectivity when receiving MT is seldom investigated, which is of importance to understand the role of MNS in MT-related neural networks. Coherence-based connectivity analysis using electroencephalography (EEG) has been used to study the focal and remote effects of NIBS (Jing and Takigawa, 2000; Jin et al., 2017). Previously, an EEG experiment has shown that observation-induced neurophysiological changes over frontal, central, and parietal areas are primarily evident in the alpha frequency band (Frenkel-Toledo et al., 2013). This finding indicated the alpha rhythm might be associated with the function of the MNS network. Therefore, the alpha frequency band has been used as the outcome to explore the neuromodulatory effects of rTMS (Jin et al., 2017) and MT (Rosipal et al., 2019) in healthy adults.

To the best of our knowledge, no study to date has explored the functional connectivity for combined effect of iTBS with MT; in existing studies, they have been applied alone. The aim of this study was to explore the combined effect of iTBS with right-hand motor training with MVF on modulating the functional connectivity at alpha frequency band during the eye closed resting state in a group of healthy adults, compared with either iTBS or MT alone. This proof-of-concept experiment would help clarify the neural network in both ipsilateral (ipsilateral to the training hand in MT) and contralateral (contralateral to the training hand in MT) hemispheres of this combined intervention and lead to future studies in the stroke population.

## Materials and Methods

### Participants

Potential participants were students enrolled from a local university via convenience sampling. Participants were invited to join the study if they could fulfill all the following inclusion criteria: (1) aged between 18 and 30; (2) right-handed, assessed by Edinburgh handedness inventory (Oldfield, 1971); and (3) normal or correct-to-normal vision. Participants were excluded if they met any of the following exclusion criteria: (1) any contraindication of NIBS (e.g., history of seizure, metal implant, current use of psychoactive drugs, etc.); (2) any known neurological or psychiatric disease; (3) any form of upper limb or hand injury in the past 3 months; and (4) upper limb or hand deformities. Written informed consent was obtained before their participation. The ethical approval for the current study was obtained from the Human Ethics Sub-Committee, University Research Committee of The Hong Kong Polytechnic University (Reference number: HSEARS20180120003). The study was designed as a controlled experiment with three parallel groups.

### Experimental Procedure

**FIGURE 1:**
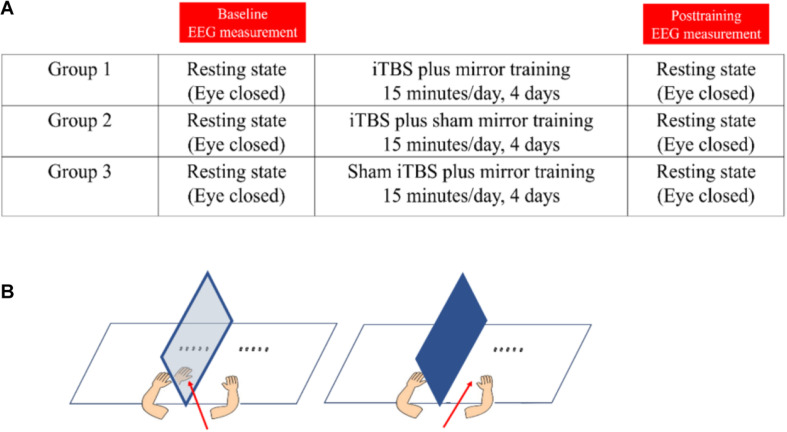
The demonstration of study design and intervention. **(A)** The study procedure. **(B)** Demonstration of mirror training (left-side) and sham mirror training (right-side). The red arrow denotes the visual direction during the training.

Participants were randomly allocated to one of the three groups: (1) iTBS plus MT, (2) iTBS plus sham MT, and (3) sham iTBS plus MT, by drawing lots. Figure 1 demonstrates the procedure of study and the intervention setup. All participants had to attend two EEG assessment sessions and four consecutive training sessions, one session per day.

### EEG Acquisition

Electroencephalography was captured with a 64-channel cap (64-channel Quik-Cap, Compumedics Neuroscan, United States) using a Digital DC EEG Amplifier and Neuroscan Curry 7 (Compumedics Neuroscan, United States). Electrode impedance was kept below 10 kohm, and signal was sampled at 1,024 Hz. A ground electrode was positioned on the forehead in front of the Cz electrode, and two reference electrodes were placed on the left and right mastoids. All channels were used during recording, although our analysis was limited to a few channels of interest. Task-designed EEG recording and corresponding results were reported in another article (Zhang and Fong, 2019). In this article, we focus on the resting state functional connectivity. Resting state EEG was recorded for approximately 2 min and 50 s at each assessment session, when the participants closed their eyes. Participants were seated upright in an electromagnetic shielded room and required to minimize any body movement during the recording. EEG data were collected by the same experimenter who performed the TBS and motor training.

### Transcranial Magnetic Stimulation Session

Standard 600-pulse iTBS protocol proposed by Huang and Rothwell (2004) (i.e., 20 trains of 10 bursts given with 8-s intervals, with a total of 600 pulses, 192 s per session) was delivered daily by MagPro stimulators (MagVenture, Denmark) with a butterfly-shape coil (C-B60), over the right motor cortex for four consecutive days. The coil was positioned at an angle of 45° to the mid-sagittal plane of the participants’ heads. The stimulation target (i.e., right motor cortex) was identified as the area in which the most consistent and largest motor-evoked potential (MEP) output was found. The positioning of the coil was maintained by means of Vicra optical tracking using the Localite neuro-navigation system based on a data set of a standard head (Localite, Bonn, Germany). The intensity of stimulation of iTBS was set at 80% of individual active motor threshold (Huang and Rothwell, 2004). Active motor threshold is defined as the minimum intensity over the motor cortex that could elicit an MEP of no less than 200 μv in five out of 10 trials during a slightly voluntary contraction (20% of the maximal voluntary contraction) of the contralateral first dorsal interosseous muscle. Sham stimulation was delivered using the same coil that delivers only 20% of the individual active motor threshold.

### Motor Training Session

Immediately after each TBS session, participants underwent right-hand motor training with a mirror or a covered mirror. We followed the previous studies using four-day MT in healthy adults (Hamzei et al., 2012; Lappchen et al., 2015). The participants in Groups 1 and 3 were required to look at the MVF reflected in the mirror when performing the right-hand motor training, with the left hand behind the mirror remaining static. Participants in Group 2 were asked to perform the same training with the mirror covered and looking directly at their moving (right) hand, the aim of which was to control the cross-education effect from the right hand motor training to the static left hand (Zult et al., 2014). Our training tasks were modified from the Nine-hole peg test, Minnesota dexterity test, Purdue Pegboard test, and two-ball rotation task, lasting for approximately 15 min per session, including picking up, placing and displacing pegs, making an assembly with a pin, a washer, and a collar, placing, displacing, and turning plastic disks, and in-hand rotation of two wooden balls. Behavioral motor performance was also assessed before and after the intervention, by using these four assessments. The results of behavioral motor performance have been reported in another article (Zhang and Fong, 2019).

### EEG Data Pre-processing

Raw EEG signals were band-pass filtered between 1 and 30 Hz, by using *pop_eegfiltnew* function in EEGLab. The data were filtered using a Hamming windowed sinc FIR filter. The filter orders were 3,300 and 440, respectively. Then the data were down-sampled at 250 Hz. By visual inspection, we rejected bad channels with abnormally high-amplitude signals and time periods containing significant movement artifacts. Channel’s data were then re-referenced to the common average. An independent component analysis algorithm was used to identify any ocular component that was further rejected (Delorme and Makeig, 2004).

Surface Laplacian transformation was carried out in order to minimize volume conduction effects from real connectivity among brain areas, *via* the current source density toolbox (Tenke and Kayser, 2005). The continuous data were segmented into several 2-s epochs before the surface Laplacian. We first generated two transformation matrices terms. The two matrices were used for the spherical spline interpolation of surface potentials (G) and current source densities (H), respectively (Perrin et al., 1989). The following parameters were used in the calculation, i.e., smoothing constant (lambda) = 10^–5^, the number of iterations = 50, *m* = 4 (the constant which affected the flexibility of the spherical splines). Then, we applied the CSD transform to EEG data with the two transformation matrices (Kayser and Tenke, 2006).

### Coherence Analysis

**FIGURE 2:**
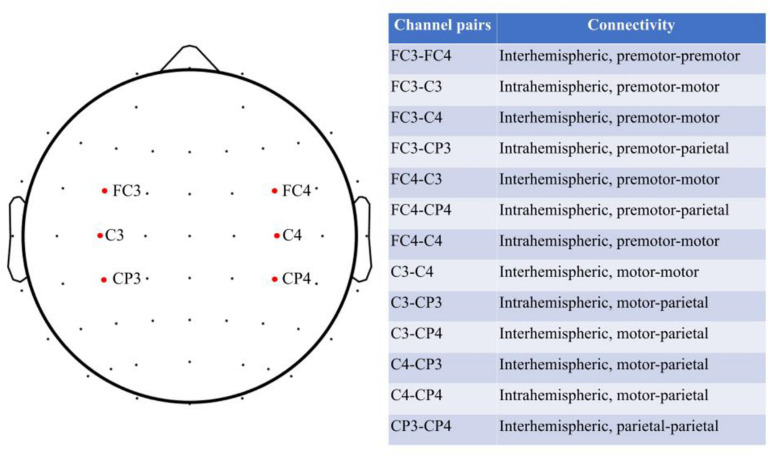
Channel pairs of interest.

Based on the previous hypothesis of MNS, we focused on the intrahemispheric connectivity within premotor, motor, and parietal areas in either left (contralateral to the training hand) or right (ipsilateral to the training hand) hemisphere, and interhemispheric connectivity among bilateral premotor, motor, and parietal areas (Zhang et al., 2018). As there is evidence showing the connectivity between ipsilateral MNS to the contralateral motor area (Hamzei et al., 2012; Saleh et al., 2017), we also explored the functional linkage of the premotor or parietal area on one side of the hemisphere with the motor area on another side of the hemisphere. Therefore, 13 pairs of channels were investigated (see Figure 2 for the selected channel pairs). Channels FC3, FC4, C3, C4, CP3, and CP4 were selected to represent the left and right premotor areas, left and right motor areas, and left and right parietal areas, respectively, according to a previous report (Jin et al., 2017). Coherence-based measure was used in the present study to probe the functional connectivity among the cortical areas (Guevara and Corsi-Cabrera, 1996). Coherence represents the normalized covariance of two time series in the frequency band. The coherence at a frequency f for signal x and y is computed by the normalization of cross spectrum as follows (Guevara and Corsi-Cabrera, 1996):

$$Coherence\left(f\right)\frac{{\left|\left\langle Pxy\left(f\right)\right\rangle \right|}^{2}}{\left(Pxx\times Pyy\right)}$$

where Pxx and Pyy refer to the auto-spectrum of signal x and y, respectively, and Pxy refers to the cross-spectral spectrum. The estimated coherence ranges from 0 to 1, in which 0 means that there is no linear dependence between the two channels at frequency f. A higher level of coherence suggests higher statistical dependence between the two signals, and vice versa. Frequency band was set at between 8 and 12 Hz, as the alpha rhythm is the dominant frequency during the eye closed resting state (Jin et al., 2017) and previous literature also shows the potential correlation between alpha rhythm and MNS activities (Frenkel-Toledo et al., 2013). Coherence was further transformed to z scores by using the following formula:

Z=0.5×log((1+coherence)/(1-coherence))

### Statistical Analysis

Statistical analysis was performed with IBM Statistical Product and Service Solutions (SPSS) version 23.0. Baseline characteristics were compared by one-way ANOVA or Fisher’s exact test. A mixed-effects regression model with random intercepts and slopes was used to detect any significant difference in the change of coherence, using the z score after log transformation, among the three groups, because of its superiority in analyzing the repeated measures data (Gueorguieva and Krystal, 2004). We included group allocation, time, and interaction of group and time as fixed-effects. The random intercept and random slope of change in dependent variables over time were included as random-effects, i.e., the model assumed that individuals differed at baseline and in the rate of change over time, which accounted for the uncontrollable variability within the sample and hence achieved greater power (Gueorguieva and Krystal, 2004). Group was labeled as 1, 2, and 3, which represented our three experimental groups. Time was coded as baseline and post-training, represented as values of 1 and 2. Maximum likelihood was chosen as the estimation method, and the heterogeneous first−order autoregressive covariance structure was selected to estimate the model parameters. Between-group differences were investigated in terms of group by time interaction effects, i.e., the difference in slope reflected the difference in change of coherence between-group (Lewthwaite et al., 2018). According to our objective, we compared the potential differences between Group 1 (iTBS plus MT) with Group 2 (iTBS plus sham MT), and Group 1 (iTBS plus MT) with Group 3 (sham iTBS plus MT). Within-group differences were examined by separated related-sample Wilcoxon signed rank tests. The level of significance was set at *p* < 0.05.

## Results

### Characteristics of Participants

We received 19 applications for participation, of which one participant was excluded due to the history of childhood epilepsy. Therefore, 18 participants were recruited for our experiment. There was no significant between-group difference in age (25.30 ± 2.00 *vs.* 26.50 ± 2.17 *vs.* 26.33 ± 2.25, *p* = 0.602) and gender (three females *vs.* two females *vs.* four females, *p* = 0.835). From the 18 participants, one case’s baseline EEG data and another case’s post-training EEG data were removed from data analysis due to significant noise. Therefore, the pre–post comparison of EEG analysis was conducted on 16 cases (Group 1 = 5 *vs.* Group 2 = 6 *vs.* Group 3 = 5). On average, 2.59 ± 2.08 channels were labeled as bad channels and thus rejected after the data preprocessing. No channel of interest was labeled as bad channels among our sample. The mean length of data that were used in the final analysis was 149.89 ± 15.57 s.

### Coherence Analysis

The results of statistical comparisons across the three groups are reported in Table 1. When comparing Group 3 and Group 1, the mixed-effect model demonstrated a significant interaction effect in the coherence between FC4 and C4 (Group 3 *vs.* Group 1, difference in slope = −0.84; SE = 0.39, *p* = 0.048), and between FC3 and FC4 (Group 3 *vs.* Group 1, difference in slope = −0.43; SE = 0.21, *p* = 0.049). The results indicated enhanced connectivity between right premotor and motor areas and bilateral premotor areas in participants from Group 1, in contrast to those in Group 3. Within-group comparison showed that there were significant differences in coherence between FC4 and C4 channels, and between C3 and C4 channels in Group 1 (Z = −2.02, *p* = 0.043).

**TABLE 1:** Results of coherence differences across three groups.

		Within-group differences		Between-group differences			
		Z	*p*	Comparisons	Δβ	SE	*p*
Pair 1: FC3-FC4	Group 1	–1.75	0.080	Group 3 *vs.* Group 1	–0.43	0.21	0.049*
	Group 2	–0.52	0.600	Group 2 *vs.* Group 1	–0.13	0.19	0.513
	Group 3	–1.21	0.225				
Pair 2: FC3-C3	Group 1	–0.67	0.500	Group 3 *vs.* Group 1	–0.19	0.50	0.715
	Group 2	–0.73	0.463	Group 2 *vs.* Group 1	0.43	0.48	0.386
	Group 3	–0.135	0.893				
Pair 3: FC3-C4	Group 1	–1.21	0.225	Group 3 *vs.* Group 1	–0.32	0.21	0.143
	Group 2	–0.94	0.345	Group 2 *vs.* Group 1	0.02	0.20	0.911
	Group 3	–0.94	0.345				
Pair 4: FC3-CP3	Group 1	–0.41	0.686	Group 3 *vs.* Group 1	–0.09	0.28	0.745
	Group 2	–1.78	0.075	Group 2 *vs.* Group 1	0.63	0.27	0.033*
	Group 3	–1.21	0.225				
Pair 5: FC4-C3	Group 1	–1.21	0.225	Group 3 *vs.* Group 1	–0.23	0.20	0.251
	Group 2	–0.11	0.917	Group 2 *vs.* Group 1	–0.21	0.19	0.277
	Group 3	–0.14	0.893				
Pair 6: FC4-C4	Group 1	–2.02	0.043*	Group 3 *vs.* Group 1	–0.84	0.39	0.048*
	Group 2	–1.36	0.173	Group 2 *vs.* Group 1	–0.10	0.38	0.784
	Group 3	–0.94	0.345				
Pair 7: FC4-CP4	Group 1	–1.21	0.225	Group 3 *vs.* Group 1	–0.25	0.18	0.170
	Group 2	–0.94	0.345	Group 2 *vs.* Group 1	–0.34	0.17	0.057
	Group 3	–0.14	0.893				
Pair 8: C3-C4	Group 1	–2.02	0.043*	Group 3 *vs.* Group 1	–0.21	0.25	0.425
	Group 2	–0.94	0.345	Group 2 *vs.* Group 1	–0.08	0.24	0.759
	Group 3	–0.41	0.686				
Pair 9: C3-CP3	Group 1	–1.48	0.138	Group 3 *vs.* Group 1	0.40	0.28	0.173
	Group 2	–1.992	0.046*	Group 2 *vs.* Group 1	0.59	0.27	0.045*
	Group 3	–0.674	0.500				
Pair 10: C3-CP4	Group 1	–0.67	0.500	Group 3 *vs.* Group 1	–0.01	0.21	1.000
	Group 2	–0.94	0.345	Group 2 *vs.* Group 1	–0.25	0.20	1.000
	Group 3	–0.41	0.686				
Pair 11: C4-CP3	Group 1	–0.41	0.686	Group 3 *vs.* Group 1	0.11	0.17	0.522
	Group 2	–1.36	0.173	Group 2 *vs.* Group 1	0.11	0.16	0.513
	Group 3	–1.75	0.080				
Pair 12: C4-CP4	Group 1	–0.67	0.500	Group 3 *vs.* Group 1	0.16	0.18	0.380
	Group 2	–1.15	0.249	Group 2 *vs.* Group 1	–0.01	0.17	0.935
	Group 3	–1.48	0.138				
Pair 13: CP3-CP4	Group 1	–1.75	0.080	Group 3 vs. Group 1	0.05	0.12	0.688
	Group 2	–1.15	0.249	Group 2 vs. Group 1	0.05	0.12	0.688
	Group 3	–1.75	0.080				

*P < 0.05.

**FIGURE 3:**
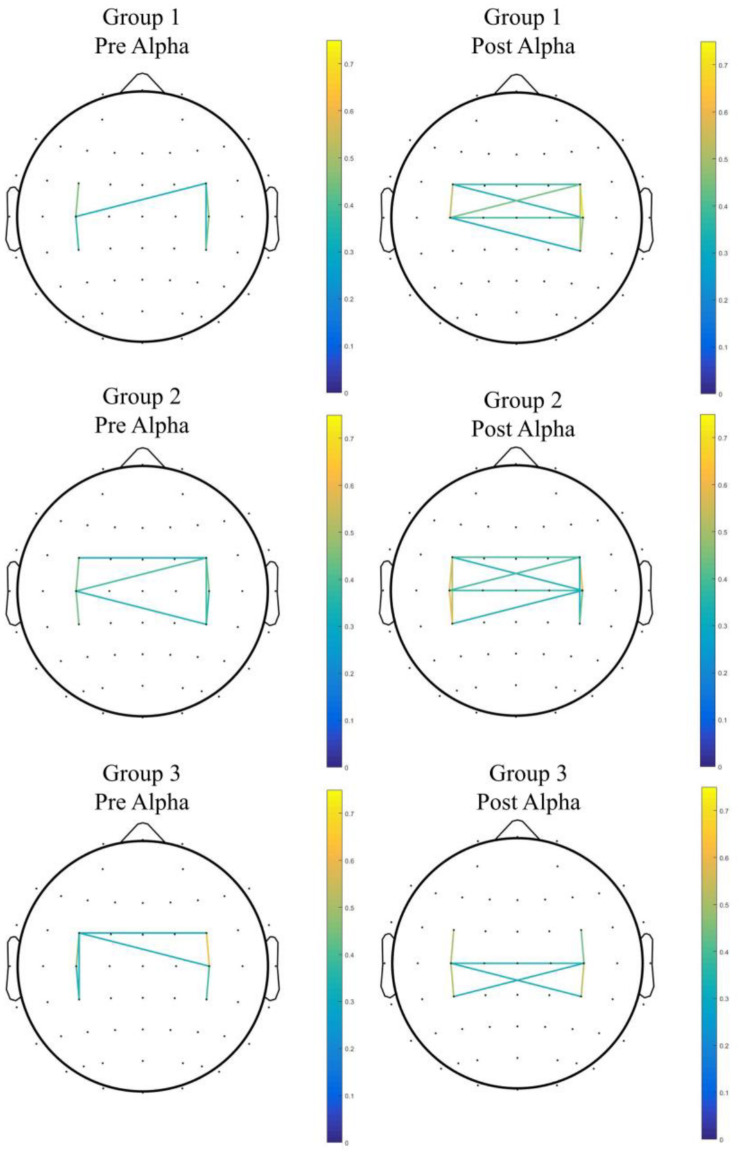
The pre–post comparison of coherence in each group. Only pairs with coherence > 0.3 were shown.

**FIGURE 4:**
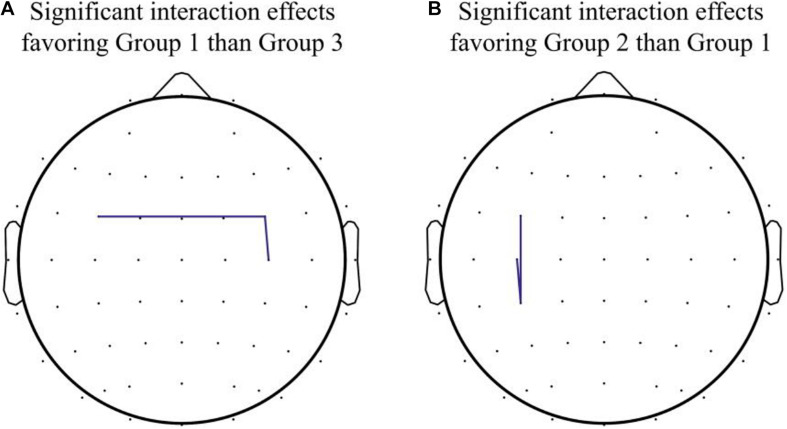
Significant interaction effects in channel pairs. **(A)** Significant interaction effects favoring Group 1 over Group 3 were noted in coherence between FC3 and FC4 and between FC4 and C4. **(B)** Significant interaction effects favoring Group 2 over Group 1 were noted in coherence between FC3 and CP3 and between C3 and CP3.

When comparing Group 2 and Group 1, the mixed-effect model demonstrated a significant interaction effect in the coherence between C3 and CP3 (Group 2 *vs.* Group 1, difference in slope = 0.59; SE = 0.27, *p* = 0.045), as well as the coherence between FC3 and CP3 (Group 2 *vs.* Group 1, difference in slope = 0.63; SE = 0.27, *p* = 0.033), which indicated enhanced connectivity among premotor, motor, and parietal areas over the contralateral hemisphere (contralateral to the moving hand and ipsilateral to the MVF) in Group 2, in contrast to Group 1. Within-group differences were found in coherence between C3 and CP3 (Z = −2.00, *p* = 0.046) in Group 2, but there was no significant interaction effect among the groups observed (see Figure 3 for the pre–post comparisons of coherence in each group and see Figure 4 for the channel pairs with significant interaction effects).

## Discussion

The present study investigated the combined effect of iTBS with MT on modulating the resting state functional connectivity at alpha frequency band, in healthy adults. Our experiment found that the combination of iTBS with MT strengthened intrahemispheric connectivity between premotor and motor areas in the ipsilateral (right) side, as indicated by the increased coherence between FC4 and C4 channels, as well as the interhemispheric connectivity of bilateral premotor cortices, as indicated by the increased coherence between FC3 and FC4 channels. Compared with participants who received iTBS with MT, those who received iTBS with sham MT showed an increase of functional connectivity between contralateral (left) premotor and parietal areas, as well as the connectivity between left premotor and motor areas, as indicated by increased coherence between FC3, C3, and CP3 channels.

The change of cortical connectivity induced by MT has been investigated in previous studies. Bai et al. (2019a) investigated the instant effect of MVF on healthy adults, using functional near-infrared spectroscopy (fNIRS) and they reported a strengthened functional correlation between the supplementary motor area and the sensorimotor area over the ipsilateral (right) hemisphere (ipsilateral to the training hand and contralateral to the MVF) when participants were viewing the MVF. Saleh et al. studied the instant effect of MVF on patients with stroke using functional magnetic resonance imaging (fMRI). Increased connectivity between contralesional inferior parietal lobule and ipsilesional primary motor cortex induced by MVF was then reported (Saleh et al., 2017). Those findings were in line with the hypothesis of MNS, which postulates that the activation of both the parietal–frontal area and the sensorimotor area could be elicited by MVF. The training effect of multiple-session MT on functional connectivity was only investigated in one previous study by Hamzei et al. (2012), which demonstrated the strengthened functional connectivity between the bilateral premotor areas and the motor area in healthy adults after four daily sessions of MT.

The effect of MT on functional connectivity in healthy adults has only been investigated by using hemodynamic signals (Hamzei et al., 2012; Bai et al., 2019a). Thus, our study probes functional connectivity in response to these two treatment modalities by EEG. The advantage of EEG is its excellent temporal resolution, which is likely to enable the detection of subtle neuroplastic changes. Compared with participants who received sham iTBS with MT, those who received iTBS with MT showed enhanced connectivity between premotor (FC4) and motor (C4) areas in the ipsilateral hemisphere as well as interhemispheric connectivity between bilateral premotor areas (i.e., FC3 and FC4). Functionally, the premotor cortex receives sensory input from the parietal cortex and projects to the motor cortex, which plays an important role in motor preparation and planning (Wong et al., 2015). There has been evidence that the premotor cortex has a mirror neuron-like property that can be activated during the observation of movement (Rizzolatti et al., 1996). A meta-analysis of fMRI experiments showed a bilateral activation of premotor areas in response to the action observation of unilateral hand movement (Caspers et al., 2010). The functional difference of left and right premotor cortices was revealed in an fMRI study, which showed that the left premotor cortex was more involved with the object manipulation during action observation and the right premotor cortex was more involved with the observed movement (Manthey et al., 2003). In a study on stroke patients, the activation of premotor cortex in both ipsilesional and contralesional hemispheres was enhanced after mirror therapy (Bhasin et al., 2012), indicating that the premotor areas on both hemispheres are likely to be part of the MNS. Strengthened connectivity among bilateral premotor and ipsilateral motor areas may indicate that the effect of MVF-based observation motor learning could be enhanced when it is in combination with iTBS over the motor cortex. A previous clinical study about the effects of MT on functional connectivity in patients with stroke, identified significant connectivity enhancement at resting state, over premotor, motor, and parietal areas bilaterally (Ding et al., 2019), in patients who received 10-session MT. Therefore, in order to yield maximal benefits for patients, there needs to be further investigation to determine an optimal dose of MT intervention when it is applied in different clinical populations with suitable priming techniques that can be combined with.

In our previous study (Zhang and Fong, 2019), we did not observe any significant difference in MVF-induced ERD and motor performance between iTBS plus MT and iTBS plus sham MT. However, using coherence analysis, we observed strengthened connectivity between left premotor, motor, and parietal areas in participants who received iTBS plus sham MT, in contrast to those who received iTBS plus MT. This finding may be attributed to the modulatory effect of active hand training with direct observation of the moving hand, which promotes the functional interrelationship among the premotor, motor, and parietal areas, but the effect was limited within the contralateral hemisphere and was not transferred interhemispherically across the corpus callosum. A previous experimental study showed that MT may have a suppression on the contralateral hemisphere (contralateral to the training hand in MT), compared with sham MT (Bartur et al., 2015). The increase in coherence in contralateral hemisphere after receiving iTBS plus sham MT might also reveal that MT may induce a shift of activation to the ipsilateral hemisphere (ipsilateral to the training hand in MT) and simultaneously suppress the activity of the contralateral hemisphere. We hypothesized that this modulatory effect to the ipsilateral hemisphere (i.e., ipsilesional hemisphere in patients with stroke) associated with MT may therefore be useful to the hemiparetic arm recovery for patients after a unilateral stroke.

## Limitations

This proof-of-concept study has several limitations. First, our current study was limited by its small sample size. Although a multiple-session intervention may stabilize the response, we cannot fully control the confounding effects in association with the inter-subject variability of iTBS. Second, we did not apply multiple comparison corrections with regard to the exploratory nature of this study. Third, EEG has its limitation in spatial resolution, although we had applied spatial filters to improve the spatial precision. Besides, our connectivity analysis was limited to coherence. This measure has been widely used to assess the functional connectivity and its meaning is easy for clinicians to understand. However, there were other novel connectivity indices that we did not use in the current study, such as phase synchronization-based measures and Granger causality-based measures. They may provide additional information about the connectivity. Lastly, we only measured the resting-state functional connectivity; motor task-related functional connectivity may also be very promising to be used as a physiological biomarker for motor recovery. In addition, different stages of movement may influence the dynamics of functional connectivity. An event-related EEG experiment with motion analysis may provide rich information for us to explore the motor task-related functional connectivity. Further study on the stroke population is needed to investigate the potential clinical effects of the combined treatments for hemiparetic arm functions.

## Conclusion

In contrast to sham iTBS combined with MT, iTBS combined with MT may strengthen the functional connectivity between bilateral premotor cortices and ipsilaterally within the motor cortex of the stimulated hemisphere. In contrast to sham MT, real MT, when combined with iTBS, might diminish the connectivity among the contralateral parietal–frontal–motor circuits, perhaps due to the shift of activation to the ipsilateral hemisphere after MT.

## Data Availability Statement

The raw data supporting the conclusions of this article will be made available by the authors, without undue reservation. Requests to access the datasets should be directed to JZ, jack-jq.zhang@connect.polyu.hk.

## Ethics Statement

The studies involving human participants were reviewed and approved by the Human Ethics Sub-Committee, University Research Committee of The Hong Kong Polytechnic University (Reference number: HSEARS20180120003). The patients/participants provided their written informed consent to participate in this study.

## Author Contributions

JZ and KF were involved in the conception and design of the study. JZ conducted the experiment and wrote up the first draft. KF supervised the progress and reviewed and edited the manuscript. Both authors approved the submission of the final version of the manuscript.

## Conflict of Interest

The authors declare that the research was conducted in the absence of any commercial or financial relationships that could be construed as a potential conflict of interest.
